# Antioxidant Compounds from Microalgae: A Review

**DOI:** 10.3390/md19100549

**Published:** 2021-09-28

**Authors:** Noémie Coulombier, Thierry Jauffrais, Nicolas Lebouvier

**Affiliations:** 1 ADECAL Technopole, 1 Bis Rue Berthelot, 98846 Nouméa, New Caledonia, France; 2 Ifremer, UMR 9220 ENTROPIE, RBE/LEAD, 101 Promenade Roger Laroque, 98897 Nouméa, New Caledonia, France; Thierry.Jauffrais@ifremer.fr; 3 ISEA, EA7484, Campus de Nouville, Université de Nouvelle Calédonie, 98851 Nouméa, New Caledonia, France; nicolas.lebouvier@unc.nc

**Keywords:** reactive oxygen species, ascorbic acid, glutathione, tocopherols, phenolic compounds, carotenoids

## Abstract

The demand for natural products isolated from microalgae has increased over the last decade and has drawn the attention from the food, cosmetic and nutraceutical industries. Among these natural products, the demand for natural antioxidants as an alternative to synthetic antioxidants has increased. In addition, microalgae combine several advantages for the development of biotechnological applications: high biodiversity, photosynthetic yield, growth, productivity and a metabolic plasticity that can be orientated using culture conditions. Regarding the wide diversity of antioxidant compounds and mode of action combined with the diversity of reactive oxygen species (ROS), this review covers a brief presentation of antioxidant molecules with their role and mode of action, to summarize and evaluate common and recent assays used to assess antioxidant activity of microalgae. The aim is to improve our ability to choose the right assay to assess microalgae antioxidant activity regarding the antioxidant molecules studied.

## 1. Introduction

The demand for natural products isolated from microalgae has increased over the last decade and has drawn attention from the food, cosmetic and nutraceutical industries. Microalgae are eukaryotic unicellular cells that combine several advantages for the development of biotechnological applications: high biodiversity, photosynthetic yield, growth, productivity and a metabolic plasticity that can be orientated using culture conditions [1,2]. Some of these metabolites are molecules of interest such as pigments (e.g., carotenoids), polyunsaturated fatty acids (PUFAs, e.g., the omega-3 or -6 fatty acids), polysaccharides, vitamins and sterols which can be introduced as dietary supplements in human nutrition and animal feed e.g., [3,4]. In addition, most of them are bioactive molecules with anti-inflammatory, antibacterial, anti-UV, antifungal, anticancer, and/or antioxidant activities which may bring added value to cosmetics, nutraceuticals or food products e.g., [5,6,7,8,9].

The demand for natural antioxidants as an alternative to synthetic antioxidants has increased [6,10]. Indeed, many synthetic antioxidants (e.g., butylated hydroxyanisole (BHA), butylated hydroxytoluene (BHT)) are considered to have a carcinogenic and/or toxic effect on animal models [11,12,13,14]. Although, most natural antioxidants currently available on the market are derived from terrestrial plants, microalgae are being more and more considered as a potential source of natural antioxidant compounds by the food industry [15,16,17] and by the cosmetic and nutraceutical industries [4,18].

Regarding the wide diversity of antioxidant compounds and mode of action combined with the diversity of ROS, this review first covers a global presentation of antioxidant molecules with their role and mode of action, to finally summarize and evaluate common and recent assays used to assess antioxidant activity of microalgae. The aim of this review is to improve our ability to choose the right assay to assess microalgae antioxidant activity regarding the antioxidant molecules studied. It also emphasizes and discusses the potential use of microalgae by the food industry for their antioxidant activity.

## 2. Antioxidant and Reactive Oxygen Species (ROS)

An antioxidant is defined as “a substance that, when present at low concentrations compared with those of an oxidizable substrate, significantly delays or prevents oxidation of that substrate” [19]. Antioxidant molecules produced by microalgae are used to protect the cell against reactive oxygen species (ROS) produced in response to biotic or abiotic stressors. Indeed, irradiance, UV, temperature, pH, metals, and nutrient can directly influence the production of antioxidant molecules in response to their availability, either through an excess or a limitation [7,20,21,22,23,24,25,26]. 

Antioxidants used for ROS detoxification have enzymatic and nonenzymatic origins with intracellular or extracellular mode of action (e.g., singlet O_2_ quencher, radical scavenger, electron donor, hydrogen donor, peroxide decomposer, enzyme inhibitor, gene expression regulation, synergist, and metal-chelating agents) [27].

In microalgae, ROS are produced by electron transport chains in chloroplasts and mitochondria, by the activity of some enzymes such as peroxidases and oxidases and also by the activity of some photosensitizers such as the chlorophyll [28]. The reactive oxygen species are therefore essentially generated in the chloroplasts and mitochondria but also in the peroxisomes [29]. More generally, ROS refer to O_2_ derivatives that are more reactive than O_2_ itself. This includes free radicals that contain at least one unpaired electron, as well as nonradical molecules [30]. Briefly, the activation of O_2_, in its stable state triplet oxygen (^3^O_2_), takes place (i) either by a transfer of energy large enough to reverse the spin of one of the electrons, which leads to the formation of singlet oxygen (^1^O_2_), or (ii) by an electron transfer that leads to the sequential reduction of ^3^O_2_ to superoxide radical (O_2_^−•^), hydrogen peroxide (H_2_O_2_) and hydroxyl radical (OH^•^). 

In plants and algae, singlet oxygen ^1^O_2_ is produced under high light by chloroplasts in the reaction center of the photosystem II (PSII) and to a lower extent in the antenna complex [31]. In the antenna complex, triplet-excited chlorophyll (^3^Chl *) is formed from singlet-excited chlorophyll (^1^Chl *) by intersystem conversion [32]. The chlorophyll in the triplet state has a longer lifespan than in the singlet state and can react with ^3^O_2_ to form the highly reactive ^1^O_2_ [33]. The singlet oxygen is responsible for extensive cell damage (e.g., protein, lipid and nucleic acid oxidation, chloroplasts and thylakoids membranes disruption and photoinhibition) around the production area [34,35]. The reaction center of PSII is thus particularly threatened. The superoxide radical (O_2_^−•^) generation takes place in the chloroplast during photosynthesis, in the mitochondria during oxidative phosphorylation and in cell membranes through the activity of the NADPH oxidase [30]. The superoxide radical is poorly reactive because it lacks the ability to modify macromolecules and is quickly transformed into hydrogen peroxide (H_2_O_2_) [34]. However, its protonated form is the precursor of much more reactive radicals [30]. The hydrogen peroxide is formed by disproportionation of the O_2_^−•^ a redox reaction that can be spontaneous or catalyzed by the superoxide dismutase (SOD). The hydrogen peroxide is also poorly reactive; however, it remains particularly toxic, as it can cross membranes, diffuse throughout the cell and oxidize sulfhydryl groups, causing the deactivation of essential enzymes [36]. It can also react with DNA and more specifically with some transition metals (e.g., iron and copper) inducing the formation of highly reactive hydroxyl radicals by the Haber–Weiss reaction [36,37]. The hydroxyl radical (OH^•^) is formed in the same cell compartments as the H_2_O_2_, i.e., in the stroma of the chloroplasts using the H_2_O_2_ generated by the photosystems, but needs the presence of reduced metal of transition [30]. The hydroxyl radicals can induce lipid peroxidation, protein and nucleic acid denaturation. In addition, there are no enzymes that can detoxify these radicals; in excess, it might lead to cell death [38], and lipid peroxidation may also generate other very reactive free radicals (e.g., the perhydroxyl HO_2_^•^, alkyl radical, reactive aldehydes malondialdehyde (MDA) and 4-hydroxy-2-nonenal (HNE)) [33,35]. Thus, the lipid-rich membranes and their functions are particularly affected by lipid peroxidation mainly through a decrease in membrane fluidity, an increase in their permeability and by enzyme, protein, ion channel and membrane receptor inactivation, which could lead to cell damage [33].

## 3. The Antioxidants Molecules of Microalgae

### 3.1. Ascorbic Acid

**Figure 1:**
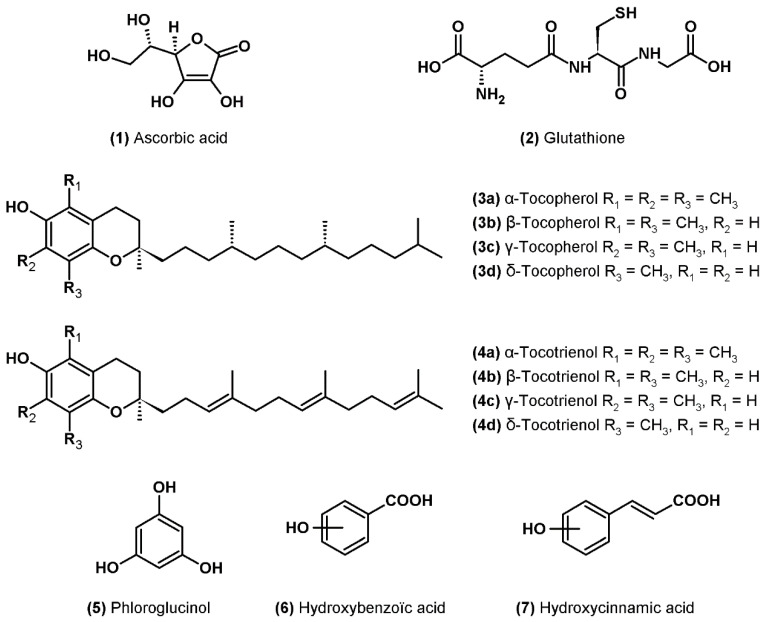
Molecular structure of ascorbic acid, glutathione, tocopherols and phenolic compounds.

Ascorbic acid or vitamin C (**1**) is one of the most abundant water-soluble antioxidants synthesized by plants (Figure 1). It is mainly present in the cytosol and chloroplasts where it can directly neutralize superoxide and hydroxyl radicals as well as singlet oxygen by electron transfer, in addition to its role in the detoxification of hydrogen peroxide during the ascorbate-glutathione cycle [39]. Ascorbic acid is also involved in the protection of the photosynthetic apparatus through its participation in the regeneration of carotenoids of the xanthophyll cycle (cofactor of violaxanthin de-epoxidase) and α-tocopherol linked to membranes [39]. It has been shown that ascorbate can also have a pro-oxidant action by the reduction of transition metals (Fe^3+^ to Fe^2+^ and Cu^2+^ to Cu^+^) which can reduce hydrogen peroxide to hydroxyl radical by the Fenton reaction [40].

### 3.2. Glutathione

Glutathione (**2**) is a water-soluble tripeptide (l-γ-glutamyl-l-cysteinylglycine) present in all cellular compartments that play a crucial role in the antioxidant response (Figure 1). In addition to its role as a cofactor in the neutralization of hydrogen peroxide by glutathione peroxidase and in the regeneration of ascorbate in reduced form via the ascorbate-glutathione cycle, glutathione can directly deactivate superoxide and hydroxyl radicals as well as singlet oxygen. In addition, like ascorbate, glutathione participates in the regeneration of α-tocopherol in its reduced form [37].

### 3.3. Tocopherols

Tocopherols or vitamin E are fat-soluble molecules only synthesized by photosynthetic organisms and located in the lipid bilayers of membranes, mainly in those of chloroplasts [41]. The name “vitamin E” groups together four natural forms of tocopherols (α-, β-, γ- and δ-) (**3a**–**d**) to which are added the four forms of tocotrienols (α-, β-, γ- and δ-) (**4a**–**d**) (Figure 1). Tocopherols and tocotrienols consist of a chromanol ring and a hydrophobic phytyl side chain, tocotrienols differing from tocopherols by the presence of three double bonds on the side chain [41].

Tocopherols and tocotrienols have the capacity to neutralize lipid peroxyl radicals by giving a hydrogen atom from the hydroxyle group of the chromanol ring, thus making it possible to stop the chain reaction of lipid peroxidation [41]. The reaction results in the formation of a hydroperoxide, which can be neutralized by the action of glutation peroxidase, and of a tocopheroxyl radical (for tocopherols) or tocotrienoxyl (for tocotrienols), which are less reactive. Tocopherols and tocotrienols can then be regenerated by the action of ascorbate and glutathione at the interface of the membrane and cytosol or by coenzyme Q (UQH_2_) in the membrane [41]. Tocopherols can also deactivate singlet oxygen by two mechanisms: a physical quenching by charge transfer and a chemical reaction resulting in the formation of tocopherol quinone by irreversible opening of the chromanol ring [42].

### 3.4. Phenolic Compounds

Phenolic compounds are a large family of molecules: more than 8000 phenolic structures have been described to date in the plant kingdom [43]. These molecules contain at least one aromatic ring carrying one or more hydroxyl groups (Figure 1). The main families of compounds are phenolic acids, tocopherols described above, flavonoids and tannins as well as stilbenes and lignans [43]. Phenolics are an important class of antioxidants in higher plants and macroalgae but have only recently been studied in microalgae. However, the total content of phenolic compounds has been shown to contribute to the antioxidant activity of microalgae extracts [10,44,45,46,47]. The main molecules identified to date in microalgae are phloroglucinol (**5**) and phenolic acids derived from hydroxybenzoic acid (**6**) and hydroxycinnamic acid (**7**). Several studies have also shown the presence of weak concentrations of flavonoids e.g., [8,47,48,49,50,51,52,53,54]. All of these molecules are found in higher plants where their concentration is generally higher than in microalgae [55].

Phenolic acids can neutralize ROS primarily by hydrogen atom transfer. The antioxidant activity of the different molecules is directly linked to their chemical structure such as the number of hydroxyl groups or their position on the aromatic cycle [55]. The reaction results in the formation of a phenoxyl radical which is stabilized by the delocalization of the single electron around the aromatic ring (resonance stabilization). Phenolic acids also have the ability to inactivate radicals by monoelectronic transfer, and some can chelate the transition metals involved in the Fenton reaction thus preventing the formation of the highly reactive hydroxyl radical [55,56].

Among the pigments, we can also mention marennine, a blue-green pigment produced by *Haslea ostrearia*, which shows particularly interesting anti-free radical and antioxidant properties [57].

### 3.5. Carotenoids

**Figure 2:**
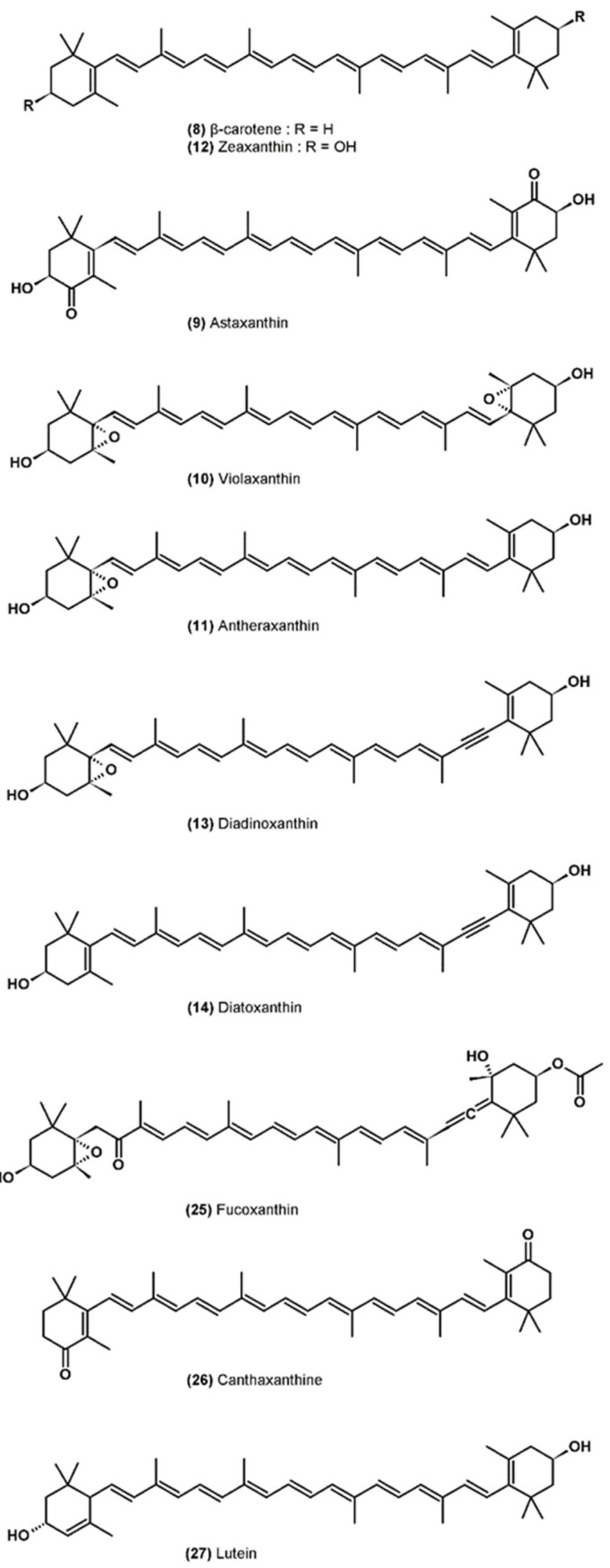
Molecular structure of carotenoids.

Carotenoids are the most common pigments in nature, and more than 750 molecules have been described in algae, higher plants, bacteria and fungi [58] (Figure 2). They are fat-soluble molecules belonging to the terpenoids family containing a central chain with a system of conjugated double bonds, which can carry cyclic end groups. Carotenoids are separated into two groups: carotenes which contain only carbon and hydrogen atoms, and xanthophylls which contain at least one oxygen atom (hydroxyl, epoxy, ketone functions, for example) [59].

Carotenoids are mainly present in the pigment-protein complexes of the thylakoid membrane, but certain species of microalgae can also accumulate carotenoids (β-carotene (**8**) and astaxanthin (**9**)) in lipid globules located in the stroma of the chloroplast or in the cytoplasm [60]. Some carotenoids are only found in specific classes of algae and so be used as chemotaxonomic markers [58].

The role of carotenoids is on the one hand to transfer light energy to chlorophylls and on the other hand to protect the photosynthetic system by deactivating ROS and preventing their formation [61]. The first photoprotection mechanism involves xanthophylls associated with the antennal complexes of the PSII which allows the dissipation of an excess of light energy without damage, according to a series of reactions called the “xanthophyll cycle” [62]. In excess light, violaxanthin (**10**) is converted to antheraxanthin (**11**) and then to zeaxanthin (**12**) by de-epoxidation provided by violaxanthin de-epoxidase, which uses ascorbate as cofactor. This enzyme, bound to thylakoids in the lumen, is activated by an acidic pH, an excess of proton in the lumen signaling that the light energy absorbed exceeds the capacity of the electron transport chain. The de-epoxidation of violaxanthin to zeaxanthin is a very rapid phenomenon on the order of a few minutes and reversible at low light intensity or in darkness by the action of zeaxanthin epoxidase. Zeaxanthin, unlike violaxanthin, can deactivate ^1^Chl* by dissipating its energy by heat [63]. This nonphotochemical quenching (NPQ) mechanism decreases the lifespan of ^1^Chl* and therefore prevents the formation of ^3^Chl* and then singlet oxygen in the PSII. In addition, by dissipating the excess energy, the possibilities of reducing O_2_ to the superoxide radical O_2_^−•^ in the PSI are minimized (less electron leakage in the transport chain) [32]. The violaxanthin cycle takes place primarily in chlorophytes. There is an alternative xanthophyll cycle, with similar photoprotective functions, in certain classes of microalgae (heterokonts, haptophytes, euglenophytes and dinophyceae) for which diadinoxanthin (**13**) is converted to diatoxanthin (**14**) [62].

At high light intensity, the probability of ^3^Chl* formation is high despite the action of the xanthophyll cycle [32]. In antenna complexes, carotenoids are located near chlorophylls and can thus quickly neutralize ^3^Chl* by triplet–triplet transfers before they react with ^3^O_2_ to form ^1^O_2_ [32]. Carotenoids can also directly deactivate singlet oxygen if it is formed [64]. This ability to deactivate ^1^O_2_ is particularly important in the reaction center of PSII where there are no carotenoids in close proximity to the special pair of chlorophylls which can change to the triplet state and then react with the ^3^O_2_ without that the reaction is not neutralized beforehand by the carotenoids [32]. Carotenoids therefore deactivate the ^1^O_2_ that is formed in the reaction center, thus protecting the photosynthetic system from oxidative damage. The deactivation of ^3^Chl* and of ^1^O_2_ results in the formation of triplet carotenoids (^3^CAR*) which de-excite without damage by dissipating the excess energy absorbed in the form of heat and can again intervene in a deactivation cycle [32].

Carotenoids are considered to be the most efficient molecules in deactivating ^1^O_2_ owing to their system of conjugated double bonds. Thus, the greater the number of conjugated double bonds is, the more effective the carotenoid will be [64]. Carotenoids also have the ability to react with free radicals through three mechanisms: hydrogen atom transfer, monoelectronic transfer and adduct formation [65].

The interactions between carotenoids and free radicals are complex. Indeed, many parameters are involved, such as the nature of the radical, the polarity of the reaction medium, the partial pressure of oxygen, the interactions with other antioxidants, such as ascorbate or tocopherols, and the concentration and structure of the carotenoid (number of conjugated double bonds, presence and types of oxygen functions, presence of end groups, cis- or trans-configuration, etc.) [65]. Carotenoids can, for example, react with a peroxyl radical (ROO^•^), which is added to the polyene chain of the carotenoid forming an adduct ROO-CAR^•^ which can react with another peroxyl radical forming a nonradical product ROO-CAR-OOR, thus allowing one to break the reaction chain of lipid peroxidation. This phenomenon takes place at low partial pressure of oxygen; however, at higher partial pressure, the ROO-CAR^•^ radical can react with ^3^O_2_ to form a ROO-CAR-OO^•^ radical which acts as a pro-oxidant and could in this case contribute to the spread of lipid peroxidation [65,66].

### 3.6. Miscellaneous Antioxidants

**Figure 3:**
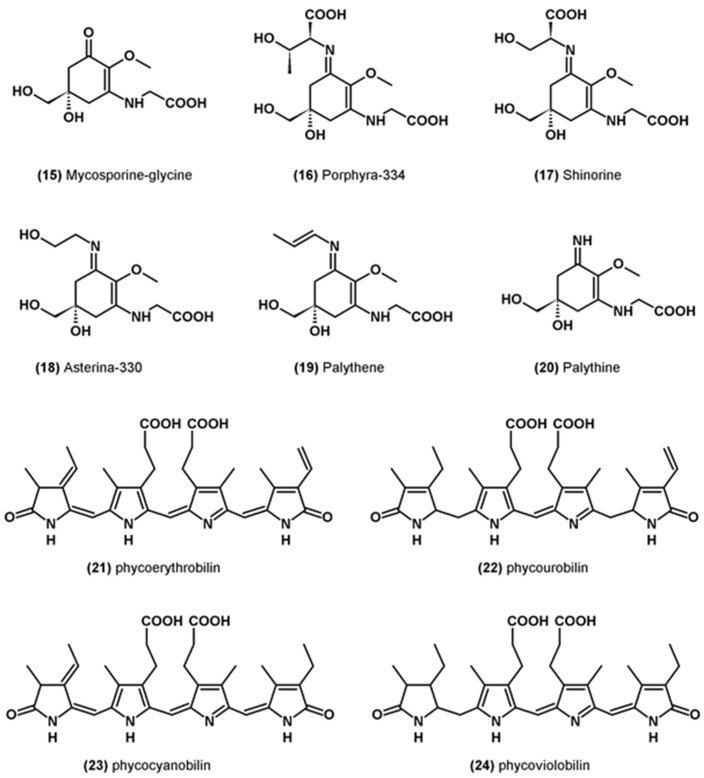
Molecular structure of other miscellaneous molecules with antioxidant activity.

There are other more specific antioxidant molecules produced by certain microalgae:

Mycosporins-like amino acids (MAA) form a family of thirty-five molecules. They are colorless, water-soluble molecules found in a wide variety of marine organisms [67]. In microalgae, the most abundant MAAs are mycosporin-glycine (**15**), porphyra 334 (**16**), shinorin (**17**), asterina-330 (**18**), palythene (**19**) and palythine (**20**) [68,69] (Figure 3). The main function of these molecules is UV protection, but some of them have also been shown to have antioxidant properties. In particular, they can inhibit lipid peroxidation and neutralize singlet oxygen and certain free radicals [67].

Polysaccharides are polymers composed of osidic units linked to glycosidic bonds attached to the cell wall or released into the medium (exopolysaccharides) [70]. Several polysaccharides derived from microalgae have shown antioxidant activity against free radicals; however, this in vitro activity remains quite low [71,72,73,74,75].

Phycobiliproteins are water-soluble pigments participating in the photosynthesis of certain groups of microalgae. They are composed of a protein and a chromophore called phycobilin particularly effective at absorbing red, orange, yellow and green light, which is not optimally absorbed by chlorophyll a [76]. There are four different structures: phycoerythrobilin (**21**), phycourobilin (**22**), phycocyanobilin (**23**) and phycoviolobilin (**24**) (Figure 3). They can neutralize ROS and chelate or reduce ferrous ions [77]. 

## 4. Common and Recent Assays Used to Evaluate Antioxidant Activity of Microalgae

Many antioxidant assays have been developed with different types of reactions to highlight the wide variety of antioxidant molecules and ROS, which act with different mechanisms. It is important to note that there is no single ideal test, and it is necessary to use several tests with different mechanisms of action to evaluate the whole antioxidant capacity of an extract or molecule [7,78,79,80].

The majority of the assays are based on the two main mechanisms of action of antioxidants (AH) to deactivate radicals (X^•^):

Hydrogen atom transfer (or HAT):

$$\mathrm{AH}+{\;X}^{•}\;\to {\;A}^{•}+\mathrm{XH}$$

These reactions are generally fast; they are completed in seconds to minutes. The effectiveness of the antioxidant is determined by its ability to give a hydrogen atom (homolytic dissociation energy); therefore, the weaker the A-H bond, the more effective the antioxidant is [80].

Single electron transfer (SET):

$$\mathrm{AH}+{\;X}^{•}\;\to {\;\mathrm{AH}}^{•+}+X^{-}$$

$${\mathrm{AH}}^{•+}\;\overset{H_{2}O}{↔}{\;A}^{•}+H_{3}O^{+}\;\;\;\;\;$$

$$X^{-}+{\;H}_{3}O^{+}\;\to \;\mathrm{XH}+{\;H}_{2}O\;$$

These reactions are slower than hydrogen transfer reactions. The reaction is pH dependent, and the effectiveness of the antioxidant is mainly determined by its ionization potential. In general, the ionization potential decreases with increasing pH leading to an increase in the ability to donate an electron by deprotonation [80]. 

Other methods can also be used to evaluate the capacity of antioxidants to chelate transition metals or to inhibit the lipid peroxidation chain reaction. The most commonly used methods to evaluate the antioxidant activity of microalgae are presented in Table 1, and the most relevant results to assess antioxidant activity of microalgae extracts by in vitro chemical methods are presented in Table 2. Some cell-based antioxidant activity assays are presented, although few results using microalgae are found in the literature (Table 3). In addition, there does not seem to be any specific assays to evaluate antioxidant activity of microalgae on an animal model. Indeed, in most cases, microalgae are administrated to animals by food with a defined period and dosage; the testing animals are then sacrificed, and common in vitro chemical antioxidant activity assays (TBARS mostly) are used on animal tissues or blood by comparing with animals that did not consume microalgae (Table 4). 

## 5. Antioxidant Activity of Microalgae

The studies included in this part have been selected using Scopus and Google Scholar databases, using the terms “microalgae” in combination with “antioxidant”, “antioxidant activity”, “antioxidant capacity” or “antioxidant properties” as keywords. The research was limited to publication with an impact factor higher than 0.5, published until 2020. The studies have been selected based on these criteria: studies using in vitro (Table 2) or in cellular assays (Table 3) reporting the antioxidant activity of crude extract of eukaryotic microalgae. Studies focusing in the antioxidant activity of specific purified metabolite(s), or antioxidant enzyme activity have not been considered. 

In addition, we have included a nonexhaustive selection of studies evaluating the antioxidant activity of microalgae in different animal models (Table 4). 

The main publications evaluating the antioxidant activity of crude microalgal extracts by in vitro chemical tests are presented in Table 2. In these studies, more than two hundred *strains* of microalgae were evaluated. The most studied genera are *Chlorella* (29 *strains*), *Scenedesmus* and *Tetraselmis* (14 *strains*) (Supplementary Materials Figure S1). 

The most commonly used assays to evaluate the antioxidant activity of microalgae are the DPPH (36 studies out of 52 referenced), ABTS (20 studies) and FCA assays (13 studies) (Supplementary Materials Figure S2). Overall, the results are very heterogeneous depending on the species of microalgae studied and the tests used to measure antioxidant activity. The protocols of the assays vary from one study to another, notably in terms of the extraction method, the solvents used, the reaction time and the concentrations tested. In addition, the results are expressed in different ways, making it difficult to compare the results. For example, for the DPPH assay, the results are expressed in percentage of inhibition for a given concentration (at different concentrations according to the studies), in IC_50_, in equivalent trolox (per unit of weight of extract or per unit of dry weight) or in equivalent ascorbic acid. Finally, the use of a reference product as a point of comparison is not systematic, and the choice of the reference product is not always relevant according to the assay used.

Nevertheless, several studies highlight the potential of microalgae as a source of antioxidants: 

*Chloromonas* sp. and *Botryidiopsidaceae* sp. (ethanolic extracts) show a strong ability to neutralize DPPH radicals (IC_50_ of 0.97 and 1.53 µg mL^−1^, respectively) and ABTS (IC_50_ of 0.95 µg mL^−1^ and 1.79 µg mL^−1^) similar to vitamin C [118,119]. The ABTS assay also revealed interesting activities of *Scenedesmus obliquus* (IC_50_ of 41 µg mL^−1^, [96]), *Haematococcus pluvialis* (activity up to 1974 µmol TE g^−1^ extract for supercritical H_2_O extraction, [114]) and *Dunaliella salina* (activity up to 1118 µmol TE g^−1^ extract with hexane extraction, [83]). Interesting results are also obtained with the DPPH assay for *Galdieria sulphuraria*, *Ettlia carotinosa*, *Neochloris texensis*, *Chlorella minutissima*, *Chlorella vulgaris*, *Schizochytrium limacinum*, *Stichococcus bacillaris* and *Crypthecodinium cohnii* with inhibition percentages between 89% and 95% with aqueous or methanolic extracts at concentrations of 250 µg mL^−1^ [106]. 

Natrah et al. [84] showed that *Chaetoceros calcitrans*, *Scenedesmus quadricauta*, *Isochrysis galbana*, *Chlorella vulgaris*, *Nannochloropsis oculata*, and *Tetraselmis tetrahele* had a strong ability to inhibit lipid peroxidation with inhibition percentages ranging from 88% to 98% for methanolic extracts at 80 µg mL^−1^ with the TBARS assay and between 88.4 and 97% for extracts at 200 µg mL^−1^ with the FTC assay (Ferric ThioCyanate assay, indirect measurement of the quantity of hydroperoxides formed during the first stages of lipid oxidation). The ability of the genera *Tetraselmis* to inhibit lipid peroxidation is confirmed by Coulombier et al. [21] who have obtained an IC_50_ up to 3,4 µg mL^−1^ with a methanol-dichloromethane extract. *Euglena tuba* also seems to be an interesting species for its ability to inhibit lipid peroxidation (IC_50_ with TBARS assay = 42 µg mL^−1^) and to neutralize the superoxide radical (IC_50_ = 5.2 µg mL^−1^, [49]). Some species show good ability to neutralize superoxide radical such as *Chaetoceros* sp. (1029 µmol TE g^−1^ dichloromethane extract), *Nannochloropsis* sp. (3224 µmol TE g^−1^ methanol extract), *Chlorella stigmatophora* and *Phaeodactylum tricornutum* (IC_50_ of 48.37 and 68.61 µg mL^−1^ with aqueous extracts, [87]). Chloroform and methanol extracts of *Chaetoceros* sp. also show interesting results with the FRAP assay (610 and 492.50 µmol TE g^−1^, [86]). Good results are also obtained with the TAC assay with IC_50_ below 100 µg mL^−1^ for methanolic extracts of *Chlorella vulgaris* and *Chlamydomonas reinhardtii* [47].

The antioxidant activity of the genus *Chlorella* has been demonstrated by several authors with different antioxidant assays. In addition to the results obtained with the DPPH, TBARS, FTC, TAC, and superoxide radical neutralization assays presented above, Aremu et al. [44,48] obtained IC_50_ up to 25 µg mL^−1^ with the β-carotene bleaching assay for *Chlorella minutissima* and *Chlorella* sp. and Plaza et al. [81] showed activities up to 1008 µmol TE g^−1^ of *Chlorella vulgaris* extract with the ORAC assay.

Overall, few links are made between these antioxidant activities and the metabolites involved. Still, correlations have been shown with carotenoid content [44,83], phenolic compound content [44,106] including flavonoids [47] and gallic acid and vitamin E content [114]. 

Despite cellular assays potentially giving more biological relevant information, as they take into account the bioavailability and metabolism of the tested compounds, we found only four studies using cellular assays to determine antioxidant activity of microalgae extract (Table 3). Those studies use different antioxidant cellular assays and different cell models (mouse fibroblast, macrophage or lymphoma cells and human liver cancer cell line).

Chloroform, methanol, acetone and 70% ethanol extracts of *Chaetoceros calcitrans* showed high nitric oxide scavenging activity in mouse macrophage with IC_50_ values of 3.46, 3.83, 15.35 and 17.94 µg mL^−1^, respectively, that is closed to reference compounds (IC_50_ of 4.7 and 6.1 µg mL^−1^ for quercetin and curcumin, [88]). This strong inhibitory activity of nitric oxide was attributed to the carotenoid content of *Chaetoceros calcitrans* (fucoxanthin, astaxanthin, violaxanthin, zeaxanthin, canthaxanthin and lutein). Karawita et al. [92] showed that *Pediastrum duplex* extract has a good protective effect against DNA damage induced by hydrogen peroxyde exposure (Comet assay). Indeed, a decrease of 80% of DNA damage on mouse lymphoma cells was measured with *Pediastrum* extract at 100 µg mL^−1^ compared to control with no microalgae extract. Good antioxidant activity was also measured with CAA (cellular antioxidant activity) and CLPAA (cellular lipid peroxidation antioxidant activity) assays on human liver cancer cell line with *Ostreopsis ovata* and *Alexandrium minutum*; however, both species extracts showed toxicity in cytotoxicity assay [91].

Similarly to cellular assays, the evaluation of the antioxidant activity of microalgae extracts by in vivo experimentations are limited compared to in vitro assays (Table 4). Those studies used different antioxidant in vitro assays couple with other physiological measurement, such as antioxidant enzyme activity, on various animal models (e.g., shrimps, chicken, catfish, rats, turbots or trouts, Table 4) to assess the effect of microalgae. The microalgae (*Schizochytrium* sp., *Chlorella vulgaris*, *Amphora coffeaformis*, *Schizochytrium limacinum*, *Acutodesmus obliquus*, *Nannochloropsis* spp., *Tetraselmis chuii* and *Botryococcus braunii*) were mostly included in the animal feed as dry microalgae with a percentage of inclusion mainly going from 1–10% or as a molecule equivalent of given antioxidant compounds. The results are variable depending on species from no effect of the microalgae tested [120,123] to a decrease in oxidative stress measurements such as the malondialdehyde or hydrogen peroxide content [124,125,126,127,128,129] or a decrease in DNA damage [123]. In most cases, it seems that the inclusion of microalgae directly in the fed has a positive effect on the animal physiology, which is promising regarding further used of microalgae in the food industry either in human or animal nutrition as functional ingredients. It also raises the question of the bioavailability of an antioxidant compound in the algal matrices and thus of the digestibility of the microalgae tested.

## 6. Applications in the Food Industry

In the food industry, antioxidants are used for human and animal nutrition as functional ingredients to provide nutritional benefits to a product (e.g., orange juice enriched with vitamin C), and as preservatives to extend the shelf life of foods and beverages to prevent their degradation by oxidation [130,131]. The use of antioxidant ingredients in food products intended for humans is highly regulated by country-specific laws owing to their potential toxicity. In the European Union, there is a list of authorized antioxidant additives, some of which may be of natural origin such as vitamin C (E300-E304), vitamin E (E306-E309), guaiac resin (E314) and rosemary extract (E392). Certain carotenoids are also authorized as dyes but can have an antioxidant role such as β-carotene (E160a), lycopene (E160d), lutein (E161b), violaxanthin (E161e), zeaxanthin (E161h), canthaxanthin (E161g) or astaxanthin (E161j) [130]. For foods and ingredients that were not significantly consumed before 1997, such as most microalgae, the "Novel Food" regulation framework was to be applied in Europe [132]. New microalgae on the market must obtain this authorization; however, to receive it, it has to be demonstrated that the product does not present any risk in terms of safety for human health [133] as some microalgae are known to produce phytotoxins [134,135,136]. In addition, and beyond the regulatory framework, to be of interest to the food industry, an antioxidant should not affect the color, smell and taste of the food and should be effective at low concentrations (0.001–0.01%), be easily usable, stable during processing and storage and be inexpensive [130,131]. The use of microalgae may thus be regarded as promising additive for human food, livestock feed and shelf life; however, it greatly depends on the microalgae productivity and nutrient compositions in protein, carbohydrates, lipids, vitamins, and antioxidants, which also strongly depend on species, mode of cultivation and culture medium composition e.g., [7,21,22]. Currently, around 10 species of microalgae or microalgae extract are authorized for human consumption in Europe as a food or food ingredient [137]. 

For livestock feed, antioxidant additives are subject to authorization before going on the market, an authorization that remains only valid ten years. On the other hand, raw materials are not subject to authorization, but a contribution of microalgae as an antioxidant in animal feed could only be considered as a raw material if it also provides proteins, minerals, fats, fibers, energy or carbohydrates [138]. Microalgae presents growth rate and dietary value of interest (e.g., polyunsaturated fatty acids, vitamins, pigments, polysaccharides) for livestock feed or aquaculture feed either fish, live feed and shellfish applications (e.g., in Table 4). Indeed, in aquaculture, the polyunsaturated fatty acids (PUFAs) eicosapentaenoic acid (DHA) and docosahexanoic acid (DHA) are of nutritional importance in aquafeeds and are hitherto ensured by inclusion of fish oil in aquafeeds. However, this resource is limited, and microalgae offer an alternative to fish oil. In addition, microalgae are not only seen as a source of PUFAs but also as source of other metabolites of interest such as pigments, polysaccharides, vitamins (e.g., vitamin E and C) and sterols which are introduced as dietary supplements for dietetic and therapeutic purposes [3,129]. In terms of applications, antioxidant molecules (asthaxanthin, lutein, β-carotene) carotenoids are produced by a wide variety of microalgae (see Table 2). 

## 7. Conclusion

Antioxidant molecules from microalgae are more and more considered as a potential source of natural antioxidant compounds by the food, the cosmetic and nutraceutical industries as they may bring benefits to their products.

However, it is very crucial to assess properly the antioxidant activity of an algal extract owing to the wide diversity of antioxidant compounds and the mode of action combined with the diversity of ROS involved. This review highlights the lack of standardization between extractions procedures used to assess antioxidant activity from microalgae matrices, and more disturbingly, it highlights the inappropriateness between the assay used and the molecules studied. These often hamper the comparison between studies and bring the authors to false or incorrect interpretation of their results. 

Therefore, although all the assays have their merits and demerits, the appropriate selection of a given assay was to be made based on the mode of action of a studied molecule in front of the principle and mechanism of an assay, especially in vitro assays. In addition to the need of normalization of the extraction procedures and to the appropriate use of an assay, we conclude that it is crucial to combine many assays to assess microalgae full antioxidant activity.

This review also highlights that microalgae are rich in antioxidant molecules with more or less potent activities, which can be used as an ingredient in food, cosmetic and nutraceutical industries. In addition, research publications are available on modern in vitro chemical methods, but application on cellular assays and in vivo experimentations are still lacking. There is a need to develop models to improve our ability to assess the activity of antioxidant molecule on these kinds of models to further improve industrial adaption and application. 

## Figures and Tables

**Table 1:** Main methods used for antioxidant activity evaluation of microalgae.

	Name of the method	Principle	Mode of Detection	Ref.
In vitro	ORAC (oxygen radical absorbance capacity) assay	measure the chain breaking capacity against peroxyl radical generated by the thermal decomposition of AAPH (2,2′-azobis (2-amidino-propane) dihydrochloride). The peroxyl radical reacts with fluorescein (fluorescent probe), causing a fluorescence loss over time	fluorimetry	[81]
	β-carotene bleaching assay	measure the inhibition capacity of β-carotene oxidation induced by radical products resulting from the peroxidation of linoleic acid. The discoloration of β-carotene is measured at 434 nm	photocolorimetry	[82]
	TEAC (trolox equivalent antioxidant capacity) assay	measure the scavenging capacity of the blue chromophore ABTS (2,2′-azino-bis (3-éthylbenzothiazoline-6-sulphonique)) radical cation, which is reduced to a colorless compound in the presence of a radical scavenger. The discoloration is followed by absorbance measure at 734 nm	photocolorimetry	[83]
	DPPH (2,2-diphenyl-1-picrylhydrazyl) radical scavenging capacity assay	measure the scavenging capacity of the purple DPPH radical which is reduced to a pale-yellow compound in the presence of a radical scavenger. The absorbance decrease is measured at 515 nm	photocolorimetry	[84]
	Reducing power assay	measure the reduction capacity of potassium ferricyanide to potassium ferrocyanide which produces a ferric ferrocyanide blue complex by reaction with ferric chloride. The absorbance of the complex is measured at 700 nm	photocolorimetry	[85]
	FRAP (ferric-reducing antioxidant power) assay	measure the reduction capacity of ferric-TPTZ (tripyridyltriazine) to ferrous-TPTZ, the latter forming a blue complex at acidic pH which is measured at an absorbance of 593 nm	photocolorimetry	[46]
	TAC (total antioxidant capacity) assay or phosphomolybdenum assay	measure the reduction capacity of molybdenum Mo(vi) to Mo(v), the latter forming a green phosphate-Mo(v) complex at low pH which is followed by absorbance measure at 695 nm	photocolorimetry	[8]
	FCA (ferrous-chelating activity) assay	measure the ferrous-chelating activity by following the formation of a magenta-colored Fe^2+^-ferrozine complex at an absorbance of 562 nm. Coexisting chelator acts as competing agents results in decrease in the absorbance	photocolorimetry	[86]
	CCA (copper-chelating activity) assay	measure the copper-chelating activity by following the dissociation of the blue complex of pyrocatechol violet (PV) with CuSO_4._ The color turned to yellow when PV dissociated a Cu ion in the presence of chelating agents. The change in color is measured at 632 nm.	photocolorimetry	[45]
	TBARS (thiobarbituric acid reactive substances) assay	measure of the end-product of lipid peroxidation which formed a pink complex with thiobarbituric acid at 100 °C in acidic condition. The formation of the complex is measured at an absorbance of 534 nm	photocolorimetry	[84]
	Superoxide radical scavenging activity assay	measure the scavenging capacity of superoxide radical generated by the reaction of NADH with phenazine methosulfate or by the oxidation of hypoxanthine by the xanthine oxidase. The inhibition of the reduction of nitroblue tetrazolium in blue-colored formazan by superoxide radical is followed at an absorbance of 560 nm.	photocolorimetry	[49,87]
	Hydrogen peroxide scavenging activity by FOX (ferrous ion oxidation–xylenol orange) assay	measure the scavenging capacity of hydrogen peroxide. Hydrogen peroxide oxidizes ferrous ion to ferric ion, which then forms a blue-purple complex with xylenol orange. The decrease in absorbance in presence of scavenger is read at 560 nm	photocolorimetry	[49]
	Hydroxyl radical scavenging activity assay	measure the scavenging capacity of hydroxyl radical which is generated by the Fenton reaction. 2-deoxyribose is oxidized by hydroxyl radical and degraded to malondialdehyde. It forms a pink complex with thiobarbituric acid at 100 °C in acidic condition which is measured at an absorbance of 532 nm.	photocolorimetry	[87]
In vitro or on cell	Nitric oxide scavenging activity assay	measure the scavenging capacity of nitric oxide (NO), generated from sodium nitroprusside. NO reacts with oxygen to produce nitrite which can be estimated by use of Griess reagent (mix of sulphanilamide, phosphoric acid and naphthylethylenediamine dihydrochloride). Scavengers of NO compete with oxygen leading to reduced production of nitrite. The absorbance of the chromophore formed by the reaction of Griess reagent and nitrite was read at 546 nm. Nitrite oxide scavenging capacity could also be evaluated with a cellular-based assay. NO release by cells is determined by measurement of nitrite concentration in culture supernatant using the Griess reagent.	photocolorimetry	[88,89]
On cell	ROS (reactive oxygen species) assay	measure the decrease in ROS produced by cells after stress induction in presence of antioxidant. The cells are incubated with the fluorescent dye CM-DCFDA (5-(e-6)-clorometil-2,7-dichloro dihydrofluorescein diacetate), and the fluorescence of the sample is measured at 535 nm (excitation 490 nm) to follow ROS production.	fluorimetry	[90]
	CLPAA (cellular lipid peroxidation antioxidant activity) assay	measure inhibition of lipid peroxidation in cellular membranes by monitoring red (590/632 nm) and green (485/520 nm) fluorescent products generated by the lipophilic probe C-11-BODIPY after addition of cumene hydroperoxide.	fluorimetry	[91]
	CAA (cellular antioxidant activity) assay	measure the inhibition of oxidation of a fluorescent probe. The nonfluorescent DCFH (2′,7′-dichlorofluorescein) is entrapped in cell and oxidized by peroxyl radical derived from ABAP (2,2′-azobis(2-amidopropane)) or AAPH decomposition producing fluorescent DCF (dichlorofluorescein). Antioxidant prevent oxidation of the probe and attenuate cellular fluorescence (excitation and emission at 485 and 520 nm)	fluorimetry	[91]
	Comet assay (single-cell gel electrophoresis)	measure the nuclear DNA protection by an antioxidant after applying hydrogen peroxide oxidative stress on cells. Treated cells are embedded in agarose and are lysed to form nucleoids containing supercoiled loops of DNA linked to the nuclear matrix. After electrophoresis, the DNA is stained with a fluorescent dye and results in structures resembling comets observed by fluorescence microscopy; the intensity of the comet tail relative to the head reflects the number of DNA breaks.	fluorescence microscopy	[92]

**Table 2:** Antioxidant activity evaluation of microalgae extracts by in vitro chemical methods (AA: ascorbic acid, AAE: ascorbic acid equivalent, ABS: absorbance, Ac: acetone, AcOH: acetic acid, AIOLA: AAPH induced oxidation of linoleic acid, BHA: butylated hydroxyanisole, BHT: butylated hydroxytoluene, CCA: copper-chelating activity, CHCl_3_: chloroform, conc.: concentration, Co-Q10: co-enzyme Q10, DCM: dichloromethane, DPPH: 2,2-diphenyl-1-picrylhydrazyl, DW: dry weight, Eq: equivalent, EtOAC: ethyl acetate, EtOH: ethanol, FA: fatty acid, FCA: ferrous-chelating activity, FRAP: ferric-reducing antioxidant power, FTC: ferric thiocyanate assay, FW: fresh weight, GC-MS: gas chromatography–mass spectroscopy, Hex: hexane, IC_50_: inhibition concentration 50, inhib.: inhibition, i-PrOH: isopropanol, MeOH: methanol, ORAC: oxygen radical absorbance capacity, PBS: phosphate buffer saline, PE: petroleum ether, PLE: pressurized liquid extraction, PUFA: polyunsaturated fatty acid, TAC: total antioxidant capacity, TBARS: thiobarbituric acid reactive substance, TE: trolox equivalent, TEAC: trolox equivalent antioxidant capacity, temp.: temperature, TPC: total phenolic compounds, US: ultrasounds, α-toco.: α-tocopherol).

Microalgae Species	Antioxidant Assay	Composition Analyses	Antioxidant Activity	Positive Control	Molecules Involved in Antioxidant Activity	Method of Extraction	Ref.
*Grammatophora marina*	(i) DPPH (ii) FCA (iii) hydrogen peroxide scavenging activity (iv) superoxide radical scavenging activity (v) hydroxyl radical scavenging activity (vi) nitric oxide scavenging activity	-	extracts at 2000 µg mL^−1^ (i) 41–86% inhib. (ii) 21–81% inhib. (iii) 14–25% inhib. (iv) 24–45% inhib. (v) 10–35% inhib. (vi) 12–33% inhib.	α-toco. and BHT at 2000 µg mL^−1^ (i) 70 and 72% inhib (ii) 10 and 11% inhib. (iii) 74 and 67% inhib. (iv) 33 and 64% inhib (v) 79 and 77% inhib. (vi) 43 and 56% inhib.	-	maceration 80% MeOH or enzymatic lysis (5 carbohydrases and 5 proteases tested)	[93]
*Chlorella vulgaris*	(i) DPPH (ii) TEAC (iii) ORAC (iv) FRAP	TPC	(i) 0.8 µmol TE g^−1^ DW (ii) 15 µmol TE g^−1^ DW (iii) 31 µmol TE g^−1^ DW (iv) 0.6 µmol TE g^−1^ DW	-	phenolic compounds	US (30 min, room temp.) EtOH 50%	[94]
*Dunaliella salina, Dunaliella tertiolecta, Phaeodactylum tricornutum, Chaetoceros muelleri, Pavlova salina, Pavlova lutheri, Tetraselmis suecica, Tetraselmis* sp., *Tetraselmis chui, Nannochloropsis* sp., *Isochrysis galbana*	ORAC	TPC, total carotenoids	45–577 µmol TE g^−1^ DW	-	-	maceration + EtOAC, Hex or H_2_O	[95]
*Scenedesmus obliquus*	(i) DPPH (ii) TEAC (iii) superoxide radical scavenging activity (iv) nitric oxide scavenging activity	carotenoids, PUFA	(i) IC_50_: 412–878 µg mL^−1^ (ii) IC_50_: 41–648 µg mL^−1^ (iii) IC_50_: 520–1236 µg mL^−1^ (iv) IC_50_ = 60 µg mL^−1^	-	-	maceration (20 min 40 °C) + EtOH, Ac, ethyl lactate or Hex/i-PrOH (3/2)	[96]
*Scenedesmus* sp. + 4 *Scenedesmus quadricauda* *strains*	(i) DPPH (ii) β-carotene bleaching	TPC, tannins, iridoids	(i) 6–70% inhib. (extracts at 200 µg mL^−1^) (ii) 24–92% inhib. (extracts at 400 µg mL^−1^)	(i) AA: 98% inhib. at200 µg mL^−1^ (ii) BHT: 70% inhib. at 400 µg mL^−1^	phenolic compounds	maceration + US (30 min, in ice) + MeOH 50%, PE or DCM	[82]
*Chlorella minutissima*	(i) DPPH (ii) β-carotene bleaching	TPC, tannins, iridoids, pigments	(i) 10–70% inhib. (extracts at 200 µg mL^−1^) (ii) IC_50_: 75–600 µg mL^−1^	(i) AA: 97% inhib. at 200 µg mL^−1^ (ii) IC_50_ BHT = 60.7 µg mL^−1^	carotenoids, phenolic compounds	maceration (1 night) + US (30 min, in ice) + MeOH, PE or DCM	[44]
*Chlorella minutissima + 2 Chlorella* sp. *strains.*	(i) DPPH (ii) β-carotene bleaching	TPC, tannins, flavonoids, iridoids	(i) 25–100% inhib. (extracts at 200 µg mL^−1^) (ii) IC_50_: 25–450 µg mL^−1^	(i) AA: 97% inhib. at 200 µg mL^−1^ (ii) IC_50_ BHT = 61 µg mL^−1^	-	maceration (1 night) + US (30 min, in ice) MeOH, PE or DCM	[48]
*Ammatoidea normanii*, *Ruttnera lamellose*, *Pavlova granifera*, *Apistonema* sp., 2 *Cryptomonas pyrenoidifera* *strains*, *Porphyridium aerugineum*, *Porphyridium sordidum*, *Audorinella* sp., *Phragmonema sordidum*, 3 *Characiopsis aquilonaris* *strains*, *Characiopsis ovalis*, 2 *Characiopsis* sp. *strains*, *Characiopsis minima*, *Pseudostaurastrum enorme*, *Goniochloris sculpta*, *Eustigmatos* sp., *Vischeria helvetica*, *Chlorobotrys gloeothece*, *Chlorobotrys* sp., *Dioxys* sp., *Coronastrum aestivale*, *Chlorella vulgaris*, *Mychonastes homosphaera*, *Gloeococcus minor*, *Pectodyction cubicum*, *Jaagiella apicola*, *Schizomeris leibleinii*, *Interfilum paradoxum*, *Micrasterias radiosa var. elegantior*, *Haematococcus pluvialis*, *Lobomonas* sp., *Stephanosphaera pluvialis*, *Bumilleria sicula*, *Euglena cantabrica*	(i) DPPH (ii) TEAC	-	(i) IC_50_: 44–1421 mg FW mL^−1^ (ii) 5–195 mg AAE 100 g^−1^ FW and 17–258 mg TE 100 g^−1^ FW	-	-	US (30 min, dark) + maceration (1 night, −4 °C) + EtOH	[6]
*Botryococcus braunii*, *Chlorella sorokiniana*, *Nannochloropsis granulata*, *Neochloropsis oleabundans*, *Phaeodactylum tricornutum*, *Porphyridium aerugineum*, *Scenedesmus obliquus*, *Scenedesmus* sp., *Tetraselmis chuii*	(i) DPPH (ii) ORAC	TPC, carotenoids, lipids, FA	(i) <50% inhib. (extracts at 200 µg mL^−1^) (ii) 7–53 µmol TE g^−1^ DW	-	phenolic compounds and lipids	maceration MeOH (DPPH) or PLE Hex/DCM (50/50)(70 °C) and then Ac/H_2_O/AcOH (70/29.5/0.5) (80 °C) (ORAC)	[97]
*Chlorella kessleri*	(i) DPPH (ii) TEAC (iii) reducing power	total carotenoids, chlorophylls *a* and *b*	(i) 1–4% inhib. (extracts at 2500 µg mL^−1^) (ii) 196–346 µmol TE g^−1^ extract (iii) ABS_700_: 0,266–0,473 (extracts at 2500 µg mL^−1^)	-	-	maceration MeOH	[98]
*Scenedesmus* sp.	(i) DPPH (ii) FRAP	TPC, flavonoids, carotenoids	(i) 0.6–3.7 µmol TE g^−1^ DW (ii) 2.8–47.0 µmol TE g^−1^ DW	-	-	US (20 min) + maceration (1 h) EtOH/H_2_O (3:1), Hex, EtOAc, or H_2_O	[99]
*Botryococcus braunii*	ORAC	-	43 µmol TE g^−1^ extract	-	-	grinding + PBS	[90]
*Euglena tuba*	(i) DPPH (ii) TBARS (iii) superoxide radical scavenging activity (iv) hydrogen peroxide scavenging activity (v) peroxynitrite scavenging activity (vi) singlet oxygen scavenging activity (vii) hypochlorous acid scavenging activity	TPC, flavonoids, tannins, alkaloids, AA	(i) IC_50_ = 146 µg mL^−1^ (ii) IC_50_ = 42 µg mL^−1^ (iii) IC_50_ = 5.8 µg mL^−1^ (iv) IC_50_ = 47340 µg mL^−1^ (v) IC_50_ = 278 µg mL^−1^ (vi) IC_50_ = 2821 µg mL^−1^ (vii) IC_50_ = 879 µg mL^−1^ (viii) IC_50_ = 223 µg mL^−1^	(i) IC_50_ AA = 5.3 µg mL^−1^ (ii) IC_50_ mannitol = 571.4 µg mL^−1^ (ii) IC_50_ quercetin = 42.1 µg mL^−1^ (iv) IC_50_ sodium pyruvate = 3.2 mg mL^−1^ (v) IC_50_ curcumin = 90.8 µg mL^−1^ (vi) IC_50_ gallic acid = 0.88 mg mL^−1^ (vii) IC_50_ lipoic acid = 0.05 mg mL^−1^ (viii) IC_50_ AA = 236.0 µg mL^−1^	-	maceration (15 h) + MeOH 70%	[49]
3 *Chlorella* sp. strain	(i) DPPH (ii) FCA (iii)TBARS	TPC	(i) IC_50_: 810–1400 µg mL^−1^ (ii) IC_50_: 1220–1500 µg mL^−1^ (iii) 5.9–88% inhib. (extracts at 4000 µg mL^−1^)	(i) IC_50_ BHT = 50 µg mL^−1^ (ii) IC_50_ EDTA = 28 µg mL^−1^ (iii) BHT 94% inhib. (conc. not specified)	-	grinding (20 min) + H_2_O 80 °C 20 min or maceration (24h) + EtOH 95%	[100]
*Nephroselmis* sp., *Tetraselmis* sp., *Dunaliella* sp., *Picochlorum* sp., *Schizochlamydella* sp., 2 *Nitzschia* sp. strain, *Thalassiosira weissflogi*, *Entomoneis punctulata*, *Cylindrotheca closterium*, *Chaetoceros* sp., *Bacillaria* sp.	(i) DPPH (ii) TEAC (iii) ORAC (iv) TBARS	carotenoids composition	(i) IC_50_ from 484 to >1000 µg mL^−1^ (ii) IC_50_ from 193 to >1000 µg mL^−1^ (iii) 0–190 µg TE mg^−1^ extract (iv) IC_50_: 15.4–473.6 µg mL^−1^ extract	(i) IC_50_ trolox = 4.7 µg mL^−1^, α-toco. = 6.2 µg mL^−1^, AA = 8.7 µg mL^−1^, β-carotene = 257.3 µg mL^−1^, astaxanthin = 228.6 µg mL^−1^ (ii) IC_50_ trolox = 6.4 µg mL^−1^, α-toco. = 10.8 µg mL^−1^, AA = 6.1 µg mL^−1^, β-carotene = 37.0 µg mL^−1^, astaxanthin = 98.5 µg mL^−1^ (iv) IC_50_ trolox = 0.2 µg mL^−1^, α-toco. = 1.3 µg mL^−1^	carotenoids	US (60 min) + MeOH/DCM (50/50)	[7]
*Nephroselmis* sp.	ORAC	carotenoids composition	63.6–154.9 µmol TE g^−1^ DW	-	carotenoids	grinding + maceration (30 min, room temp., dark) + EtOH	[22]
*Tetraselmis* sp.	TBARS	-	IC_50_: 3.4–11.3 µg mL^−1^ extract	IC_50_ trolox = 0.2 µg mL^−1^, IC_50_ α-toco. = 1.3 µg mL^−1^	-	grinding + US (10 min., ice bath, dark) + MeOH/DCM (50/50)	[21]
*Tetraselmis chuii*, *Nannochloropsis oculata*, *Chlorella minutissima*, *Rhodomonas salina*	(i) DPPH (ii) FCA (iii) CCA	TPC	extracts at 1000 µg mL^−1^ (i) 0–21% inhib. (ii) 12–98% inhib. (iii) 12–22% inhib.	conc. at 1000 µg mL^−1^ (i) BHT: 88% inhib. (ii) EDTA: 95% inhib. (iii) EDTA: 74% inhib.	-	grinding + maceration (1 nuit) + Hex or MeOH	[45]
*Isochrysis galbana T-iso*, *Tetraselmis* sp., *Scenedesmus* sp.	(i) DPPH (ii) FCA (iii) CCA	TPC, FA	(i) IC_50_ > 1000 µg mL^−1^ (ii) IC_50_: 730–4110 µg mL^−1^ (iii) IC_50_: 900 µg mL^−1^ to >10000 µg mL^−1^	(i) IC_50_ BHT = 70 µg mL^−1^ (ii) IC_50_ EDTA = 100 µg mL^−1^ (iii) IC_50_ EDTA = 280 µg mL^−1^	-	grinding + Hex, and, Ac and H_2_O in sequential order	[101]
*Chlorococcum minutum*	(i) TAC (ii) reducing power	TPC	(i) 2.5–10 mg AAE g^−1^ extract (ii) 1–4 mg AAE g^−1^ extract	-	phenolic compounds	maceration (72 h) EtOH, MeOH, or Ac	[102]
*Chaetoceros calcitrans*	(i) DPPH (ii) TEAC (iii) FCA	TPC, major phenolic compounds, total carotenoids totaux, fucoxanthin	(i) 0.1–1.4 mg TE g^−1^ DW (ii) 1.2–10.6 mg TE g^−1^ DW (iii) 0.3–18.5 mg Na-EDTA Eq g^−1^ DW	-	carotenoids and phenolic compounds	grinding + US (30 min, room temp.) + MeOH, EtOH, Ac, Ac 90%, Ac/CHCl_3_ (90/10) or Ac/CHCl_3_/MeOH (80/10/10)	[103]
*Chaetoceros calcitrans*, *Isochrysis galbana*, *Skeletonema costatum*, *Odontella sinensis*, *Phaedactylum tricornatum*	(i) TEAC (ii) FRAP (iii) FCA (iv) β-carotene bleaching	TPC, major phenolic compounds, total carotenoids totaux, fucoxanthin	(i) 2.0–21.5 mg TE g^−1^ DW (ii) 0.2–2.0 mg TE g^−1^ DW (iii) 1.5–13.4 mg EDTA eq g^−1^ DW (iv) 0.1–1.4 mg TE g^−1^ DW	-	carotenoids and phenolic compounds	grinding + MeOH	[104]
*Chaetoceros* sp., *Nannochloropsis* sp.	(i) DPPH (ii) FRAP (iii) FCA (iv) superoxide radical scavenging activity	TPC	(i) 14.0–106.7 µmol TE g^−1^ extract (ii) 171.5–609.8 µmol TE g^−1^ extract (iii) 3.2–82.4 µmol EDTA Eq g^−1^ extract (iv) 227.9–3224.5 µmol TE g^−1^ extract	-	-	maceration (24 h) + Hex, DCM, CHCl_3_ or MeOH	[86]
*Nannochloropsis oculata*, *Nannochloropsis* sp., *Isochrysis* sp., *Isochrysis* ISO-T, *Tetraselmis* sp., *Tetraselmis suecica*, *Botryococcus braunii*, *Porphyridium cruentum*, *Neochloris oleabundans*, *Chaetoceros calcitrans*, *Chlorella vulgaris*, *Haematococcus pluvialis* (red and green phase), *Parachlorella kessleri*, *Phaeodactylum tricornutum*, *Schizochytrium* sp.	(i) TEAC (ii) FRAP (iii) AIOLA	TPC, total carotenoids	(i) 0–69 µmol TE g^−1^ DW (ii) 3.3–90 µmol TE g^−1^ DW (iii) 1.8–89.7 µmol TE g^−1^ DW	-	carotenoids and phenolic compounds	grinding + maceration (30 min) + EtOH/H_2_O (3/1) or Hex, EtOAc and H_2_O (80 °C) in sequential order	[10]
*Phaeodactylum tricurnutum*, *2 Chlorella vulgaris strains*, *Haematococcus pluvialis*, *Scenedesmus maximus*, *Scenedesmus obliquus*, *Scenedesmus quadricauda*, *Desmodesmus pleimorphus*, *Nannochloropsis* sp., *Pavlova lutheri*, *Porphyridium aerugineum*	TEAC	carotenoids	0.8–149 mg L^−1^ AAE µg^−1^ chlorophyll *a*	-	-	grinding + EtOH 50%	[105]
*Galdieria sulphuraria*, *Ettlia carotinosa*, *Neochloris texensis*, *Chlorella minutissima*, *Stichococcus bacillaris*, *Schizochytrium limacinum*, *Crypthecodinium cohnii*, *Chlorella vulgaris*	DPPH	TPC	89–95% inhib. (extracts at 250 µg mL^−1^)	BHT: 98% inhib. at 250 µg mL^−1^	TPC	US (20 min) MeOH or maceration H_2_O (100 °C, 30 min)	[106]
*Chlorella stigmatophora*, *Phaeodactylum tricornutum*	(i) Superoxide radical scavenging activity (ii) hydroxyl radical scavenging activity (iii) hypochlorous acid scavenging activity	-	(i) IC_50_: 48–170 µg mL^−1^ (ii) IC_50_: 180–250 µg mL^−1^ (iii) IC_50_ > 1000 µg mL^−1^	-	-	US + H_2_O then soxhlet + DCM and MeOH on extraction residue	[87]
*Chlorella vulgaris*	FRAP	TPC	0.01–58.2 µmol TE g^−1^ DW	-	phenolic compounds	maceration + Hex, EtOAc + H_2_O (80 °C) in sequential order	[46]
*Phaeodactylum tricornutum*, *Nannochloropsis gaditana*, *Nannochloris* sp., *Tetraselmis suecica*	(i) DPPH (ii) reducing power (iii) FCA	TPC, flavonoids, carotenoids	(i) IC_50_: 356–400 µg mL^−1^ (ii) 24–33 AAE mL^−1^ (iii) IC_50_: 2810–12820 µg mL^−1^	(i) IC_50_ AA = 3,7 µg mL^−1^ (ii) BHT = 1,4 AAE mg^−1^ (iii) IC_50_ EDTA = 10 µg mL^−1^	-	Not specified	[51]
*Dunaliella salina*	TEAC	carotenoids	11–1118 µmol TE g^−1^ extract	-	carotenoids	PLE Hex, EtOH or H_2_O	[83]
*Dunaliella salina*	TEAC	carotenoids	115–452 µmol TE g^−1^ extract	-	carotenoids	sub- and super-critical CO_2_	[107]
*Chlorella vulgaris*, *Chlamydomonas reinhardtii*	(i) DPPH (ii) TAC (iii) FRAP	TPC, flavonoids	(i) IC_50_: 397–423 µg mL^−1^ (ii) IC_50_: 55–73 µg mL^−1^ (iii) ABS_700_: 0.136 to 0.124 (extracts at 250 µg mL^−1^)	(ii) IC_50_ AA = 127.5 µg mL^−1^ (iii) ABS_700_ AA = 0.423 at 250 µg mL^−1^	flavonoids	maceration MeOH	[47]
*Ankistrodesmus* sp., *Euglena cantabrica*	DPPH	-	8–71% inhib. (extracts at 1000 µg mL^−1^)	conc. at 1000 µg mL^−1^ BHT: 26% inhib., BHA: 91% inhib.	-	maceration (40 min) + MeOH or H_2_O	[108]
*Halochlorococcum porphyrae*, *Oltamannsiellopsis unicellularis*	(i) DPPH (ii) FCA (iii) hydrogen peroxide scavenging activity (iv) superoxide radical scavenging activity (v) hydroxyl radical scavenging activity (vi) nitric oxide scavenging activity	TPC	extracts at 2000 µg mL^−1^ (i) 42–95% inhib. (ii) 4–72% inhib. (iii) 5–42% inhib. (iv) 5–58% inhib. (v) 4–31% inhib (vi) 1–51% inhib.	conc. at 2000 µg mL^−1^ (i) BHT and α-toco: 94% inhib. (ii) BHT: 11% inhib., α-toco 10% inhib. (iii) BHT 60% inhib., α-toco 62% inhib. (iv) BHT 63% inhib., α-toco 61% inhib. (v) BHT 77% inhib., α-toco 79% inhib. (vi) BHT 26% inhib., α-toco 25% inhib.	-	80% MeOH then fractionation with Hex, CHCl_3_ and EtOAc or enzymatic lysis (5 carbohydrases and 5 proteases tested)	[89]
*Chlamydomonas nivalis*, *Chlorella protothecoides*, *Chlorella pyrenoidosa*, *Chlorella vulgaris*, *Chlorella zofingiensis*, *Crypthecodinium cohnii*, *Nitzschia laevis*, *Schizochytrium* sp., *Schizochytrium mangrovei*, *Thraustochytrium* sp.	TEAC	TPC	0–11.4 µmol TE g^−1^ DW	-	-	maceration (30 min) + Hex, EtOAc and H_2_O (80 °C) in sequential order	[13]
*Tetraselmis* sp., *Dunaliella salina*, *Dunaliella* sp., *Nannochloropsis gaditana*, *Chlorella* sp., *Navicula* sp., *Phaeodactylum tricurnutum*, *Chaetoceros* sp., *Isochrysis* sp.	DPPH	TPC, total carotenoids, PUFA	IC_50_: 247–464 µg mL^−1^	IC_50_ BHT = 6.2 µg mL^−1^, IC_50_ AA = 2.5 µg mL^−1^	-	maceration (3h, dark) + EtOH	[109]
*Isochrysis galbana*	(i) DPPH (ii) TEAC	TPC, β-glucan, Co-Q10, β-carotene, fucoxanthin	(i) 0–17 mg AAE L^−1^ (ii) 52–56 µmol TE g^−1^ DW	-	-	grinding + maceration (18 h) EtOH 96% or H_2_O	[110]
*Nannochloropsis gaditana*	(i) DPPH (ii) β-carotene bleaching (iii) FRAP	carotenoids, tocopherols, FA	(i) 1,1–1,8 µmol TE g^−1^ extract (ii) 64–97% inhib. (extracts at 1000 µg mL^−1^) (iii) 48–86 µmol Fe(II) g^−1^ extract	-	carotenoids, tocopherols, FA	Supercritical CO_2_	[111]
*Dunaliella salina*, *Oocystis pusilla*, *Scenedesmus rubescens*	DPPH	TPC	0.4–17.5 µmol TE g^−1^	-	phenolic compounds	maceration (30 min, 25 °C) + Hex, EtOAc and H_2_O (80 °C) in sequential order	[112]
*Cymbella* sp., *Navicula* sp., *Skeletonema costatum*, *Isochrysis galbana*, *Chaetoceros calcitrans*, *Nannochloropsis oculata*, *Tetraselmis tetrathele*, *Scenedesmus quadricauda*, *Chlorella vulgaris*, *Oocystis* sp., *Trachelomonas* sp.	(i) DPPH (ii) FTC (iii)TBARS	-	(i) no activity for extracts at 250–1000 µg L^−1^ (ii) 0–97% inhib.(extracts at 200 µg mL^−1^) (iii) 0–98% inhib. (extracts at 80 µg mL^−1^)	(i) α-toco: 85% inhib., quercetin: 65% inhib, BHT: 74% inhib. (100 µg L^−1^) (ii) α-toco: 84% inhib, quercetin: 92% inhib., BHT: 100% inhib. (200 µg mL^−1^) (iii) α-toco: 71% inhib., quercetin: 90% inhib., BHT: 98% inhib. (80 µg mL^−1^)	-	maceration (4 j) + MeOH	[84]
2 *Nannochloris* sp. *strains*, *Picochlorum* sp., *Desmochloris* sp.	(i) DPPH (ii) FCA (iii) CCA	TPC, pigments	extracts at 1000 µg mL^−1^ (i) <10% inhib. (ii) <25% inhib. (iii) <30% inhib.	conc. at 1000 µg mL^−1^ (i) BHT: 88% inhib. (ii) EDTA: 96% inhib. (iii) EDTA: 76% inhib.	-	grinding + maceration (1 night, 20 °C) + MeOH	[113]
*Chlorella vulgaris*	(i) TEAC (ii) ORAC (iii) superoxide radical scavenging activity	TPC	(i) 146–789 µmol TE g^−1^ extract (ii) 243–1008 µmol TE g^−1^ extract (iii) IC_50_: 8260–10752 µg mL^−1^	rosemary extract (i) 2805–2811 µmol TE g^−1^ (ii) 4615–4892 µmol TE g^−1^ (iii) IC50: 464–665 µg mL^−1^	-	supercritical H_2_O	[81]
*Haematococcus pluvialis*	TEAC	GC-MS	366–1974 µmol TE g^−1^ extract	-	α-toco., gallic acid, caramelization products and possible Maillard reaction products	supercritical H_2_O	[114]
*Phaeodactylum tricornutum*, *Nannochloropsis salina*, *Nannochloropsis limnetica*, *Chlorella sorokiniana*, *Dunaliella salina*, *Desmodesmus* sp.	(i) DPPH (ii) TEAC (iii) FCA (iv) FRAP (v) TAC	TPC, flavonoids, phenolic acids, tocopherols, carotenoids composition	(i) 8–14% inhib. (extracts at 250 µg mL^−1^) (ii) 2.7–24.2 TE g^−1^ (iii) 3–9% chelation (extracts at 250 µg mL^−1^) (iv) 0.1–0.5 AAE g^−1^ (v) 3.0–8.9 gallic acid Eq g^−1^	-	phenolic compounds, carotenoids and tocopherols	US (45 min in the dark at room temp.) + MeOH	[8]
*Tetraselmis suecica*	DPPH	pigment composition	21.1% inhib. (extract at 50 µg mL^−1^)	α-toco: 6% inhib. at 50 µg mL^−1^	-	maceration (30 min in the dark under nitrogen atmosphere at room temp.) + EtOH/H_2_O (3/1)	[115]
*Parachlorella kessleri*	(i) DPPH (ii) TEAC (iii) FCA (iv) TAC	TPC, chlorophyll *a* and *b*, total carotenoids	(i) 32–69% inhib. (extracts at 100 µg mL^−1^) (ii) 1.4–3.0 µmol TE g^−1^ extract (iii) 20% inhib. (extracts at 500 µg mL^−1^) (iv) 2.2–4.3 mg AAE g^−1^ extract	-	-	grinding + maceration MeOH	[116]
*Trentepohlia umbrina*	(i) DPPH (ii) reducing power (iii) superoxide radical scavenging activity	TPC, flavonoids	(i) IC_50_ = 665.3 µg mL^−1^ (ii) ABS_700_ = 0.0124(extract at 125 µg mL^−1^) (iii) IC_50_ = 838.8 µg mL^−1^	(i) IC_50_ AA = 6.4 µg mL^−1^ (ii) ABS_700_ AA = 0.0478 at 125 µg mL^−1^ (iii) IC_50_ AA = 115.6 µg mL^−1^	-	maceration (72 h) + MeOH	[85]
*Dunaliella salina*	DPPH	chlorophylls, total carotenoid	15–57% inhib. (extract at 250 µg mL^−1^)	AA: 95% inhib. at 250 µg mL^−1^	-	US (10 min) + maceration (4 j) + EtOH	[117]
*Skeletonema marinoi*	TEAC	TPC, flavonoids, AA, β-carotene, diatoxanthin	250–1500 fg AAE cell^−1^	-	phenolic compounds, flavonoids, AA	US (1 min, in ice) + maceration (30 min, dark) MeOH	[53]
*Chloromonas* sp.	(i) DPPH (ii) TEAC	-	(i) IC_50_ = 1.0µg mL^−1^ (ii) IC_50_ = 0.9 µg mL^−1^	(i) IC_50_ AA = 0.1 µg mL^−1^ (ii) IC_50_ AA = 0.2 µg mL^−1^	-	maceration (24 h) + EtOH	[118]
*Botryidiopsidaceae* sp.	(i) DPPH (ii) TEAC	-	(i) IC_50_ = 1.5 µg mL^−1^ (ii) IC_50_ = 1.8 µg mL^−1^	(i) IC_50_ AA = 0.2 µg mL^−1^ (ii) IC_50_ AA = 0.2 µg mL^−1^	-	maceration (24 h) + EtOH	[119]
*Crypthecodinium cohnii*, *Schizochytrium* sp.	(i) DPPH (ii) TAC (iii) FCA (iv) reducing power	TPC, flavonoids	extracts at 500 µg mL^−1^ (i) 15–30% inhib. (ii) ABS_695_: 0,500–1,000 (iii) 10–60% inhib. (iv) ABS_700_: 0,050–0,300	(ii) BHT: ABS_695_ = 0,500 at 500 µg mL^−1^ (iii) EDTA: 65% inhib. at 50 µg mL^−1^ (iv) BHT: ABS_700_ = 0,300 at 500 µg mL^−1^	phenolic compounds	maceration (2 j) EtOH 70%	[54]

**Table 3:** Antioxidant activity evaluation of microalgae extracts by cellular assays (AA: ascorbic acid, Ac: acetone, CAA: cellular antioxidant activity, CHCl3: chloroform, CLPAA: cellular lipid peroxidation antioxidant activity, EtOH: ethanol, Hex: hexane, IC50: inhibition concentration 50, inhib.: inhibition, MeOH: methanol, NMR: nuclear magnetic resonance, PBS: phosphate buffer saline, ROS: reactive oxygen species, TPC: total phenolic compounds, US: ultrasounds).

Microalgae Species	Antioxidant Assay	Composition Analyses	Antioxidant Activity	Positive Control	Molecules Involved in Antioxidant Activity	Extraction Method	Ref.
*Chaetoceros calcitrans*	Nitric oxide scavenging activity assay on RAW 264.7 cells (mouse macrophage)	metabolites profiling by ^1^H NMR + TPC	IC_50_: 3.5–187.7 µg mL^−1^	IC_50_ quercetin = 4.7 µg mL^−1^ IC_50_ curcumin = 6.1 µg mL^−1^	Fucoxanthin (25), astaxanthin, violaxanthin, zeaxanthin, canthaxanthin (26), and lutein (27)	US (30 min, room t °C) + MeoH, 70% EtOH, Ac, CHCl_3_ or Hex	[88]
*Botryococcus braunii*	(i) ROS assay and (ii) Comet assay on NIH3T3 cells (mouse embryonic fibroblast cells)	-	extract at 0.1–0.05% (i) reduction of ROS production of 35% over the control (=no microalgae extract) after stress induction (ii) no activity	(i) AA: reduction of ROS production by 64% over the control at 250 µM	-	crushing in PBS + silica sand	[90]
*Pediastrum duplex*, *Halochlorococcum porphyrae*, *Oltmannsiellopsis unicellularis*, *Achnanthes longipes*, *Navicula* sp., *Amphora coffeaeformis*	Comet assay on L5178 cells (mouse lymphoma cells)	Crude lipid content	extract at 25–100 µg mL^−1^ (i) inhibitory effect to DNA damage until 80% over the control (=no microalgae extract) after stress induction	-	-	Enzymatic extraction by 5 carbohydrases and 5 proteases	[92]
*Cylindrotheca closterium*, *Coscinodiscus actinocyclus*, *Nitzschia closterium*, 2 *Pseudo-nitzschia pseudodelicatissimastrains*, *Tetraselmis suecica*, *Isochrysis galbana*, *Skeletonema costatum*, *Lauderia annulata*, *Leptocylindrus danicus*, *Chaetoceros affinis*, *Odontella mobiliensis*, *Leptocylindrus aporus*, *Thalassiosira rotula*, *Thalassiosira weissflogii*, 2 *Skeletonema marinoistrains*, *Thalassiosira rotula*, *Skeletonema costatum*, *Stephanopyxis turris*, *Bacteriastrum hyalinum*, *Guinardia striata*, *Proboscia alata*, *Guillardia theta*, *Rhodomonas baltica*, *Rhinomonas reticulata*, *Alexandrium tamutum*, *Alexandrium andersonii*, *Ostreopsis ovata*, *Alexandrium minutum*, *Lepidodinium viride*, *Prorocentrum gracile*	(i) CAA and (ii) CLPAA on HepG2 cells (human liver cancer cell line)		extract at 50 µg mL^−1^ (i) 66–70% inhib. for *Ostreopsis ovata* (ii) 61–74% inhib. for *Ostreopsis ovata* and 100% inhib. for *Alexandrium minutum* but both species showed toxicity in cytotoxicity assay	-	-	US (1 min)+ H_2_O then addition of Ac + maceration (50 min, room temp.) then fractionation on Amberlite XAD16N resin	[91]

**Table 4:** Antioxidant activity evaluation of microalgae extracts by in vivo experimentations (CAT: catalase, DNPH: 2,4-dinitrophenyl hydrazine, FA: fatty acid, FRAP: ferric-reducing antioxidant power, GPX: glutathione peroxidase, GSH: reduced glutathione, MDA: malondialdehyde, PX: peroxidase SOD: superoxide dismutase, TAC: total antioxidant capacity, and TBARS: thiobarbituric acid reactive substance).

Microalgae Species	Experimental Animals	Concentration of Microalgae Tested	Experimental Time	Antioxidant Assay	Other Measure	Activity	Ref.
*Schizochytrium* sp.	Pacific white shrimps (*Litopenaeus vannamei*)	0–75 g of dry microalgae kg^−1^ of feed	12 weeks	TBARS on tail muscle	antioxidant enzymes activity (CAT, SOD), lipid composition of food and muscle	No effect of microalgae	[120]
*Chlorella vulgaris* and *Amphora coffeaformis*	Chickens (Cobb 500 broiler chick)	1 g of dry microalgae kg^−1^ of feed	32 days	TBARS on breast meat	SOD activity, FA and amino acids profiles of microalgae	28–31% decrease in MDA compared to control group (feed without microalgae)	[121]
*Schizochytrium limacinum*	Chickens (Arbor Acres chick)	1–2% of dry microalgae in feed	42 days	In breast and thigh muscle (i) TAC (ii) TBARS	antioxidant enzymes activity of serum (SOD, GPX, CAT), FA composition of diet and muscle	Compared to control group (feed without microalgae): (i) 33–81% increase in TAC (ii) 11–35% decrease in MDA content	[122]
*Acutodesmus obliquus*	Catfish (*Rhamdia quelen*)	1–3% of residual microalgae biomass (after oil extraction) in feed	60 days	(i) TBARS in liver (ii) Comet assay in erythrocytes, liver, and brain	Antioxidant enzymes activity (SOD, CAT), pigment determination of microalgae residual biomass	(i) No effect of microalgae (ii) Decrease in DNA damage with 3% of microalgae in erythrocytes and liver, no effect in brain tissue	[123]
*Nannochloropsis gaditana*	Normal and diabetic Wistar rats	10% of dry microalgae in feed	8 weeks	(i) TBARS of liver mitochondria and liver tissue (ii) DNPH (protein oxidation) on liver mitochondria and liver tissue	On microalgae biomass: total carotenoids, carbohydrates, total lipids and total protein On liver mitochondria and tissue: antioxidant enzymes activity (SOD, CAT, GSH)	Compared to control group (feed without microalgae): (i) Normal rats: 0–8% decrease in MDA content Diabetic rats: 35% decrease in MDA content (ii) Normal rats: no effect. Diabetic rats: 18–25% decrease in protein oxidation	[124]
*Nannochloropsis* sp.	Juvenile turbots (*Scophthalmus maximus* L.)	2.5–10% of dry microalgae in feed	10 weeks	(i) TBARS in serum and liver (ii) TAC	Antioxidant enzyme activity (SOD, GPX) in serum and liver	Compared to control group (feed without microalgae): (i) 19–56% decrease in MDA content, (ii) 9–44% increase in TAC	[125]
*Tetraselmis chuii*	Pacific white shrimps postlarvae (*Litopenaeus vannamei*)	25–100% of dry microalgae in feed	12 days	In shrimp tissue (i) hydrogen peroxide content (ii) TBARS	Proximate analysis and antioxidant activity of the feed	(i) Decrease of about 0–25% of hydrogen peroxide content (ii) No effect of microalgae on lipid peroxidation	[126]
*Haematococcus pluvialis*, *Botryococcus braunii*	Wistar rats	Administration by intubation to the stomach of a single dose of one of the two microalgae biomass solubilized in olive oil as source of 200 µM equivalent of astaxanthine or lutein	9 h	TBARS in plasma and liver	Analysis of carotenoids from plasma, liver and eyes Antioxidant enzyme activity (SOD, CAT, PX) in plasma and liver	25–61% decrease in MDA content compared to MDA content at t_0_	[127]
*Haematococcus pluvialis*, *Botryococcus braunii*	Wistar rats	Administration of a daily dose of one of the two microalgae biomass solubilized in olive oil as source of 200 µM equivalent of astaxanthine or lutein	15 days	TBARS in plasma and liver	Analysis of carotenoids from plasma, liver and eyes Antioxidant enzyme activity (SOD, CAT, PX) in plasma and liver	45–64% decrease in MDA content compared to MDA content at t_0_	[128]
*Haematococcus pluvialis*	Juvenile rainbow trout (*Oncorhynchus mykiss*)	1–10 g of dry microalgae kg^−1^ of feed	30 days	In serum (i) FRAP (ii) TBARS	alkaline phosphatase, alanine aminotransferase, aspartate and serum total protein, glucose, triglycerides, and cholesterol	Compared to control group (feed without microalgae): (i) 36–75% increase in activity (ii) 44–69% decrease in MDA content	[129]

## Supplementary Materials

The following are available online at https://www.mdpi.com/article/10.3390/md19100549/s1, Figure S1. Top 15 microalgae genus studied for their antioxidant activity, Figure S2. In vitro chemical assays used to evaluate the antioxidant activity of microalgae crude extracts.
