# Supplementary material for: Antioxidant Compounds from Microalgae: A Review
Source: Mar Drugs. 2021 Sep 28;19(10):549. doi: 10.3390/md19100549 (PMC8537667; doi:10.3390/md19100549)
Supplement: Supplementary file 1 [file marinedrugs-19-00549-s001.zip › marinedrugs-1400000-supplementary.pdf]

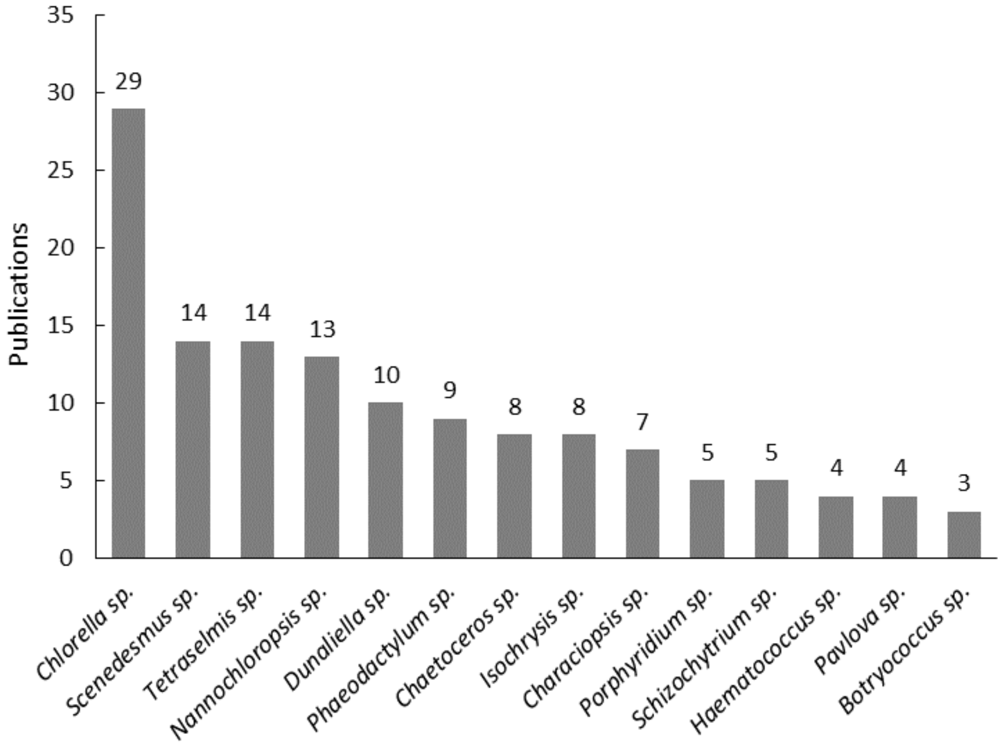

Figure S1. Top 15 microalgae genus studied for their antioxidant activity

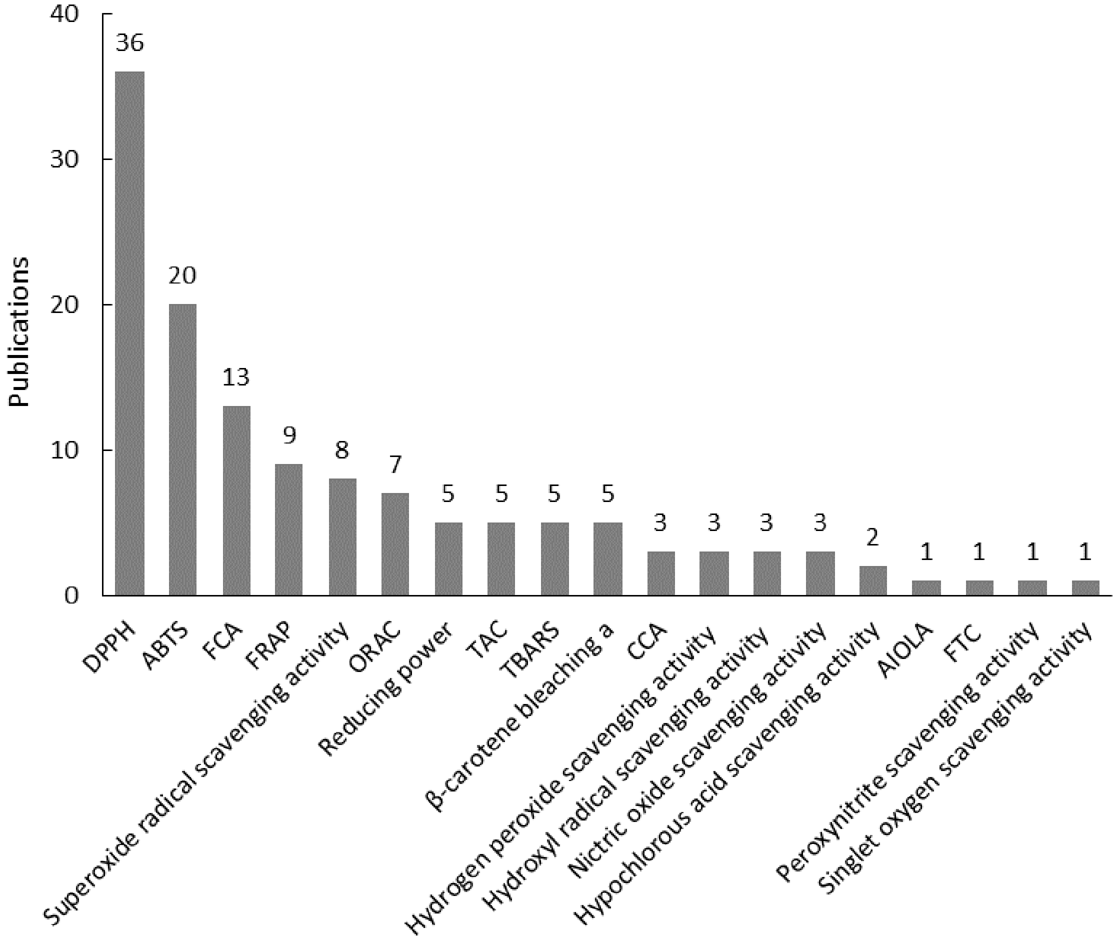

Figure S2. In vitro chemical assays used to evaluate the antioxidant activity of microalgae crude extracts
